# A Familial Thoracic Aortic and Arterial Aneurysm Syndrome Associated With FBN2 (Y1311C) and MYH11 (R34T) Variants: A Multigenerational Case Report

**DOI:** 10.7759/cureus.103596

**Published:** 2026-02-14

**Authors:** Akhtar Purvez, Ana Mir, Mudhasir Bashir

**Affiliations:** 1 Clinical Research, Momentum Medical Research, Charlottesville, USA; 2 Clinical Sciences, Lincoln Memorial University-DeBusk College of Osteopathic Medicine, Harrogate, USA; 3 Clinical Sciences, Momentum Medical Research, Charlottesville, USA; 4 Psychiatry and Behavioral Sciences, University of Virginia, Charlottesville, USA

**Keywords:** aortic root dilation, familial aortopathy, fbn2 mutation, heritable thoracic aortic disease, myh11 mutation

## Abstract

Heritable thoracic aortic disease (HTAD) is a group of genetic conditions that make people more likely to have problems with their thoracic aorta, such we talk about a rare family where a 64-year-old man had a stroke caused by a tear in a brain artery, which led to worsening thoracic aortic disease that needed surgery to replace his aortic valve and root, treatment for peripheral artery aneurysms, and later, a pacemaker for heart issues. Genetic testing found the same fibrillin-2 (FBN2) (Y1311C) change in the father and both of his sons, and the father and younger son also had a different change, myosin heavy chain 11 (MYH11). Screening imaging showed that both sons had mild aortic root dilation. This case highlights an uncommon familial aortopathy involving overlapping extracellular matrix and smooth muscle contractile pathways and illustrates the value of cascade genetic testing and longitudinal imaging surveillance of at-risk family members.

## Introduction

Heritable thoracic aortic disease (HTAD) refers to inherited conditions that weaken the aorta and increase the risk of aneurysm formation and arterial dissection. Thoracic aortic aneurysm and dissection are major contributors to cardiovascular morbidity and mortality worldwide [1]. Recognition of an underlying heritable aortopathy has significant implications for patient management, risk stratification, and family screening. Expansion of existing registries or development of new, dedicated registries is essential to better define the clinical presentation, natural history, and long-term outcomes of aneurysm syndromes [2].

A growing number of genes have been implicated in thoracic aortic aneurysm and dissection, underscoring the genetic heterogeneity of these conditions. Genetic testing enables identification of pathogenic variants that confer increased risk for aneurysm formation, arterial dissection, or both, and may help guide decisions regarding surveillance intensity and the optimal timing of prophylactic aortic repair [3]. Collectively, heritable thoracic aortic disease accounts for a substantial proportion of non-atherosclerotic thoracic aortic pathology, particularly among younger individuals and those with multisite arterial involvement. Variants affecting extracellular matrix integrity and vascular smooth muscle contractility contribute to a broad phenotypic spectrum characterized by variable expressivity and incomplete penetrance [4].

Advances in cardiovascular genetics have broadened recognition of heritable aortopathies beyond classic syndromic disorders. Pathogenic variants in genes related to microfibrillar architecture and smooth muscle function may present predominantly with vascular manifestations, often in the absence of distinctive skeletal or ocular features. As a result, genetically mediated aortic disease may remain unrecognized until the occurrence of arterial dissection or aneurysmal complications.

Among the genes associated with heritable thoracic aortic disease, variants in fibrillin-2 (FBN2) are more commonly linked to congenital contractural arachnodactyly, with vascular involvement reported less frequently, while myosin heavy chain 11 (MYH11) variants are classically associated with smooth muscle dysfunction and familial thoracic aortic aneurysm and dissection. The coexistence of variants affecting both extracellular matrix structure and smooth muscle contractility is uncommon and may contribute to more complex or variable vascular phenotypes. This case is notable for the multigenerational presence of an FBN2 variant with partial co-segregation of a MYH11 variant and differing degrees of vascular involvement within the same family.

Early identification of heritable aortopathy facilitates individualized surveillance strategies, informs surgical decision-making, and enables cascade testing of first-degree relatives. In this report, we describe a multigenerational family with variants in FBN2 and MYH11, highlighting intrafamilial phenotypic variability and the clinical relevance of overlapping pathogenic mechanisms.

## Case presentation

All clinical details presented here pertain exclusively to the authors’ own patient and are derived from primary medical records. The proband is a 64-year-old male who experienced an ischemic cerebrovascular accident in year one secondary to right middle cerebral artery dissection, resulting in persistent neurologic deficits. He subsequently developed chronic unilateral pain and motor impairment consistent with central post-stroke sequelae.

In year two, transthoracic echocardiography demonstrated aortic root dilation. Longitudinal imaging surveillance revealed progression of thoracic aortic disease, and in year three, cross-sectional imaging identified ascending aortic ectasia and bilateral common iliac artery aneurysms. In year five, the patient underwent combined aortic valve and root replacement with a bioprosthetic valve.

In year nine, the patient experienced a transient ischemic attack manifesting as amaurosis fugax without new imaging abnormalities. In year 10, bilateral iliac artery aneurysms were treated with endovascular stent placement, and later that year, symptomatic bradycardia necessitated implantation of a leadless pacemaker.

Given the constellation of arterial dissection, thoracic and peripheral aneurysmal disease, and family history of aortic pathology, genetic testing was pursued. The proband and both sons were found to carry the FBN2 Y1311C variant. The proband and younger son additionally carried the MYH11 R34T variant. Screening echocardiography demonstrated mild aortic root dilation in both sons.

This genetic testing was performed using a clinical cardiovascular gene panel. The FBN2 Y1311C variant was reported by the testing laboratory as a variant of uncertain significance with supporting evidence based on conservation, in silico prediction, and prior reports of vascular involvement in FBN2-associated conditions. The MYH11 R34T variant was similarly classified as a variant of uncertain significance. Aortic measurements were obtained using standard echocardiographic and cross-sectional imaging techniques, with longitudinal surveillance performed according to institutional protocols for heritable thoracic aortic disease.

**Figure 1:**
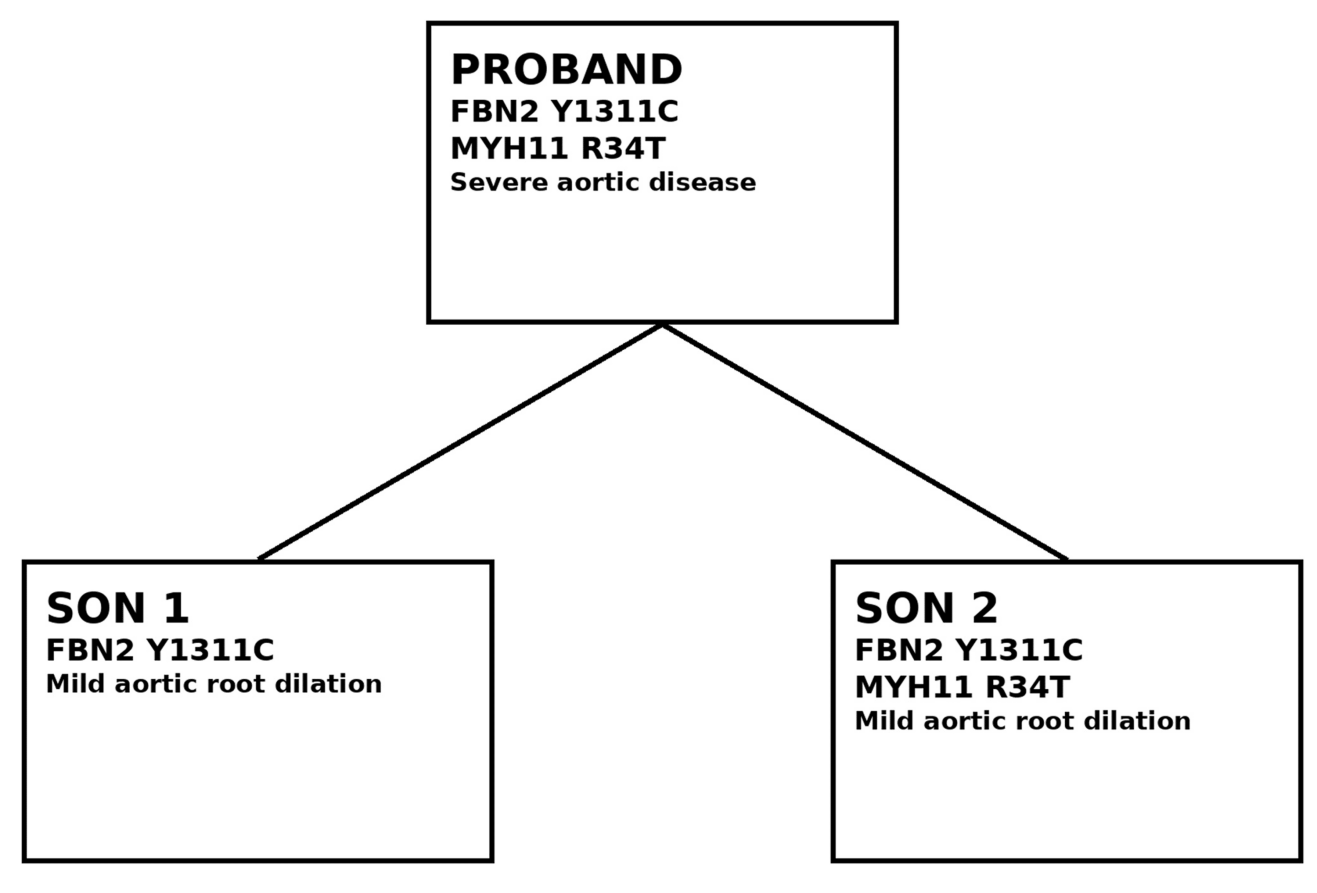
The multigenerational pedigree with segregation of the FBN2 Y1311C and variant across the proband and sons. FBN2: fibrillin-2; MYH11: myosin heavy chain 11.

Table 1 summarizes the year-by-year timeline of the proband’s major vascular and genetic events. Figure 1 illustrates the multigenerational pedigree of the family, demonstrating segregation of the FBN2 Y1311C variant in the proband and both sons, with additional MYH11 R34T co-segregation in the proband and younger son.

**Table 1:** Proband timeline of vascular and genetic events. FBN2: fibrillin-2; MYH11: myosin heavy chain 11.

Year	Event
Year 1	Right middle cerebral artery dissection with ischemic stroke
Year 2	Aortic root dilation recognized
Year 3	Ascending aortic ectasia (4.5 cm) and bilateral iliac artery aneurysms (R 2.5 cm, L 2.3 cm)
Year 5	Aortic valve and root replacement with bioprosthetic valve
Year 6	Genetic testing: FBN2 (Y1311C) in proband and both sons; MYH11 (R34T) in proband and younger son
Year 9	Transient ischemic attack presenting as amaurosis fugax; antiplatelet therapy initiated
Year 10	Bilateral iliac artery stent placement
Year 10	Leadless pacemaker implantation for bradycardia

Current clinical guidelines emphasize genetic evaluation and systematic imaging of first-degree relatives when heritable thoracic aortic disease is suspected, even in the absence of classic syndromic features [2,3].

**Figure 2:**
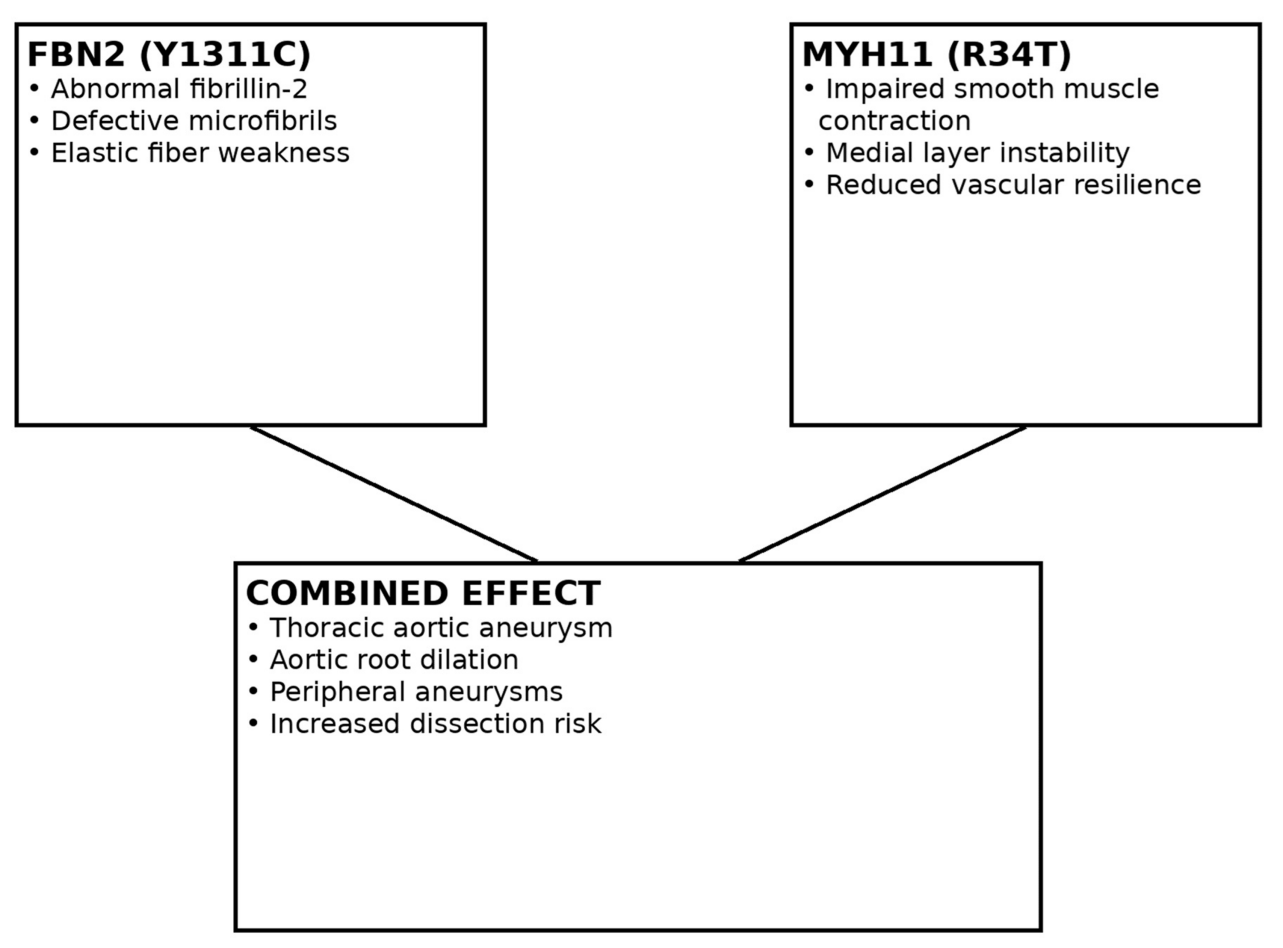
A conceptual model illustrating overlapping pathogenic mechanisms associated with FBN2 and MYH11 variants. FBN2: fibrillin-2; MYH11: myosin heavy chain 11.

Figure 2 provides a conceptual model illustrating overlapping pathogenic mechanisms associated with FBN2 and MYH11 variants. The diagram highlights how disruption of extracellular matrix microfibrillar integrity and impaired vascular smooth muscle contractility may converge to weaken the arterial wall, predisposing to thoracic aortic dilation, aneurysm formation, and arterial dissection.

Table 2 summarizes phenotypic features reported in association with FBN2- and MYH11-related aortopathies, including thoracic aortic aneurysm, aortic root dilation, arterial dissection, and peripheral aneurysms. The table contextualizes the family’s clinical presentation within the broader reported disease spectrum.

**Table 2:** Phenotypic features observed in this family within the broader spectrum of FBN2- and MYH11-associated aortopathies. FBN2: fibrillin-2; MYH11: myosin heavy chain 11.

Phenotypic feature	FBN2 (approx.)	MYH11 (approx.)	Selected references
Thoracic aortic aneurysm	Reported	Reported	[5,6]
Aortic root dilation	Reported	Reported	[5,7]
Arterial dissection	Rarely reported	Reported	[6,8]
Peripheral aneurysms	Uncommon	Reported	[6,8]
Variable expressivity/incomplete penetrance	Common	Common	[7,9]

The key clinical features in this case included early arterial dissection, progressive dilation of the thoracic aortic root and ascending aorta, and the development of peripheral aneurysms involving the iliac arteries. The presence of multisite vascular disease, combined with a family history of aortic root dilation in first-degree relatives, represented an important early indicator of a possible heritable aortopathy. Cascade genetic testing confirmed a shared FBN2 variant among affected family members, with additional MYH11 co-segregation in the proband and one son, illustrating intrafamilial variability in vascular expression.

## Discussion

This multigenerational family illustrates a rare form of heritable thoracic aortic disease characterized by a shared FBN2 Y1311C variant and partial co-segregation of MYH11 R34T. The proband demonstrated severe, multisite vascular involvement, while both sons exhibited milder but clinically relevant aortic root dilation identified through cascade screening. This pattern highlights the marked intrafamilial variability and incomplete penetrance that are characteristic of genetically mediated aortopathies [7,8]. Recognition of overlapping and less common aortopathy genotypes is increasingly important as multigene testing becomes integrated into routine cardiovascular practice [9].

Variants affecting FBN2 disrupt fibrillin-2, a critical component of extracellular matrix microfibrils required for elastic fiber assembly and arterial wall integrity [5]. Separately, pathogenic variants in MYH11 impair vascular smooth muscle cell contractile function, predisposing affected individuals to thoracic aortic aneurysm, arterial dissection, and related vascular anomalies [6]. The coexistence of variants affecting both extracellular matrix structure and smooth muscle contractility may help explain the proband’s more aggressive vascular phenotype compared with his affected offspring.

The overlapping biological mechanisms associated with FBN2 and MYH11 variants provide a conceptual framework for understanding how combined defects in microfibrillar architecture and smooth muscle function can converge to weaken the arterial wall. Such convergence likely increases susceptibility to progressive aortic dilation, aneurysm formation, and arterial dissection, even in the absence of classic syndromic features.

The phenotypic manifestations observed in this family are consistent with the broader spectrum reported in association with FBN2- and MYH11-related aortopathies. Thoracic aortic aneurysm, aortic root dilation, arterial dissection, and peripheral aneurysms have all been described with variable frequency and severity across reported cohorts, reflecting substantial heterogeneity in clinical expression [7-9].

Current clinical guidelines emphasize the importance of genetic evaluation and systematic imaging of first-degree relatives when heritable thoracic aortic disease is suspected, even in the absence of overt syndromic features [2,3]. More detailed reporting of variant classification criteria, imaging thresholds, and longitudinal measurements may further improve reproducibility and facilitate genotype-phenotype correlation in future studies of rare or overlapping aortopathy syndromes. As multigene testing becomes increasingly integrated into routine cardiovascular practice, recognition of overlapping and less common aortopathy genotypes will be essential for accurate risk stratification, individualized surveillance, and prevention of life-threatening vascular complications [9].

## Conclusions

This case report talks about a rare condition affecting multiple generations of a family, linked to the FBN2 Y1311C and MYH11 R34T gene changes, which caused serious blood vessel problems in the main patient and widening of the differences seen within the family, where the main person had serious vascular issues and their close relatives had less severe but still important aortic dilation, show that heritable thoracic aortic disease can affect people in different ways. Importantly, this family demonstrates that clinically significant vascular pathology may occur even in the absence of classic syndromic features, which points to the importance of heightened clinical suspicion in patients presenting with arterial dissection, aneurysmal disease, or a relevant family history.

The findings emphasize the need to think about heritable thoracic aortic disease in patients with arterial dissection and multiple aneurysms, especially when standard cardiovascular risk factors do not fully explain their condition. Cascade genetic testing and systematic imaging of close family members helped identify those at risk early and guided personalized monitoring plans before serious symptoms or dangerous vascular problems occurred. As genetic testing becomes increasingly integrated into routine cardiovascular practice, recognition of overlapping and less common aortopathy genotypes, such as combined fibrillin and smooth muscle contractile pathway involvement, will be essential for accurate risk stratification, personalized longitudinal monitoring, timely intervention, and the prevention of life-threatening vascular complications across affected families.
